# Energy-Efficient Algorithm for Broadcasting in *Ad Hoc* Wireless Sensor Networks

**DOI:** 10.3390/s130404922

**Published:** 2013-04-12

**Authors:** Naixue Xiong, Xingbo Huang, Hongju Cheng, Zheng Wan

**Affiliations:** 1 School of Information Technology, Jiangxi University of Finance and Economics, Nanchang 330013, China; E-Mails: nxiong@coloradotech.edu (N.X.); cloudcity66@yahoo.com.cn (Z.W.); 2 School of Computer Science, Colorado Technical University, Colorado Spring, CO 80907, USA; 3 College of Mathematics and Computer Science, Fuzhou University, Fuzhou 350108, China; E-Mail: huangxingbo1987@163.com

**Keywords:** wireless sensor networks, minimum connected dominating set, minimum-energy broadcast problem, network lifetime maximization

## Abstract

Broadcasting is a common and basic operation used to support various network protocols in wireless networks. To achieve energy-efficient broadcasting is especially important for *ad hoc* wireless sensor networks because sensors are generally powered by batteries with limited lifetimes. Energy consumption for broadcast operations can be reduced by minimizing the number of relay nodes based on the observation that data transmission processes consume more energy than data reception processes in the sensor nodes, and how to improve the network lifetime is always an interesting issue in sensor network research. The minimum-energy broadcast problem is then equivalent to the problem of finding the minimum Connected Dominating Set (CDS) for a connected graph that is proved NP-complete. In this paper, we introduce an Efficient Minimum CDS algorithm (EMCDS) with help of a proposed ordered sequence list. EMCDS does not concern itself with node energy and broadcast operations might fail if relay nodes are out of energy. Next we have proposed a Minimum Energy-consumption Broadcast Scheme (MEBS) with a modified version of EMCDS, and aimed at providing an efficient scheduling scheme with maximized network lifetime. The simulation results show that the proposed EMCDS algorithm can find smaller CDS compared with related works, and the MEBS can help to increase the network lifetime by efficiently balancing energy among nodes in the networks.

## 1. Introduction

Recent advances in micro-electro-mechanical systems, digital electronics and wireless communications have led to the emergence of *ad hoc* wireless sensor networks, which consist of a large number of sensing devices that are capable of sensing, processing and transmitting environmental information. The wireless sensor networks are expected to be connected to the ubiquitous cloud environment in future networks. In this new paradigm, smart devices will collect data, relay the information or context to each another, and process the information collaboratively using cloud computing and similar technologies. One of the most crucial issues in the wireless sensor networks is energy efficiency, because battery replacement/recharge of sensor nodes is generally difficult or even impossible in many application scenarios, such as the cases huge number of sensor nodes are placed in hostile or remote environments.

In *ad hoc* wireless sensor networks, it is a popular operation to send one message from one identified source node to all other nodes. Such an operation is generally called broadcasting, and can be used in various scenarios, *i.e.*, network topology discovery processes, network configuration processes, routing processes, and so on. In addition, broadcast operations in wireless sensor networks are generally different from those in wired networks because multiple nodes can be reached by a single transmission without any additional cost on the sender side. Generally, in the case where a message is sent from the source to all nodes, it can be generally described as a broadcast tree in which the source node acts as the tree root and the other nodes have their separate parents, and the non-leaf nodes shall relay and forward the message to their children after reception.

To provide an efficient solution for the broadcast problem with consideration of energy consumption is an important issue in wireless sensor networks. As mentioned, sensor nodes are generally supported with limited energy budget, and energy efficiency is an important issue in wireless sensor networks. Radio is a main source of energy consumption in wireless sensor networks, which is generally comprised with three parts, namely, transmission power, reception power and idle power, and the idle power is small enough to be ignored compared with the other two [1]. Since all nodes in the network shall receive messages when the broadcast process is finished, energy consumption is accordingly determined by energy cost during the transmission process. In case that the transmission range is identical for nodes in the network, the minimum-energy broadcast problem is converted to the problem of finding a spanning tree with a minimized number of non-leaf nodes.

An efficient way to build a spanning tree with a minimum number of non-leaf nodes is using the minimum Connected Dominating Set (CDS) for a given a connected network graph. Nodes in minimum CDS shall be connected and have to relay the received message, while other nodes shall have at least one neighbor located in the CDS. In this work, we have introduced an efficient heuristic algorithm to build the Minimum CDS (EMCDS). The basic idea is that we first select the Maximal Independent Set (MIS) with the help of a proposed ordered sequence list; then we build the connected dominating set by adding connected nodes into MIS; and finally we further optimize the result by removing some redundant non-leaf nodes.

The proposed EMCDS algorithm aims at providing a broadcast tree with minimum energy consumption for one single broadcast operation. However, because non-leaf nodes consume more energy compared with leaf nodes, the non-leaf nodes might become energy depleted in the broadcast operation goes on for a long time. This might result in single-node failures or network partition problems in case that the reserved energy cannot support the required operation. Sensor nodes can be carefully scheduled to balance energy consumption during the broadcast process. In this work, we have introduced a Minimum Energy-consumption Broadcast Scheme (MEBS) to avoid the node failure problem and aimed at providing an efficient scheduling scheme with the network lifetime maximized. The proposed algorithm uses a modification version of EMCDS with energy considered, and selects different relay nodes in a dynamic manner to improve the network lifetime.

The rest of this paper is organized as follows: in Section 2 we summarize related works. Section 3 describes our EMCDS algorithm and Section 4 concerns the proposed MEBS algorithm. Experimental results are presented in Section 5, while conclusions are drawn in Section 6.

## 2. Related Works

The minimum-energy broadcast problem in wireless sensor networks has received significant attention over the last few years. Here, we will introduce the key mechanisms and characteristics of the broadcast algorithms that are among the most representative of this research area.

Flooding is the simplest and most basic method for broadcast operation, but can lead to the known broadcast storm problem [2]. The broadcast storm causes severe contention and collisions among nodes, and finally results in low network performance. Many flooding mechanisms concerned with the broadcast storm problem have been proposed. In [3] the authors tried to suppress the rebroadcast of duplicate packets based on some basic network information such as location, retransmission probability, or the number of duplicate packets received by each node. The authors of [4] proposed a dynamic probabilistic approach for broadcasting. They used the packet counter in counter-based approaches to adjust the probability of forwarding. In case that one node was located in a dense area, it could receive a large amount of rebroadcasts from its neighbors, and thus the calculated packet counter was rather high; accordingly this node shall decrease the re-broadcast probability. Hanashi *et al.* [5] proposed another dynamic probabilistic approach, which set the value of the rebroadcast probability for every host node according to its neighbor's information. Jeong *et al.* [6] proposed an adaptive broadcasting method that utilized the neighbor type information. The neighbor nodes were divided into three types: parent (upper level), sibling (same level), and child (lower level) nodes. Generally, the more siblings a node had, the higher probability it had that its child nodes received a broadcast packet, although it did not retransmit the packet immediately. In case that one node had a lot of sibling nodes, its retransmission probability would be decreased. Meanwhile, in case that one node had many child nodes, the node had to retransmit a broadcast packet with a higher probability because all the child nodes were very unlikely covered by one single retransmission.

In many works, the broadcast problem is converted to the problem of finding a spanning tree. One notable contribution was the Broadcast Incremental Power (BIP) algorithm proposed by Wieselthier *et al.* [7]. It was based on “node-based” nature of wireless communications rather than the traditional “linked-based” approach. The main objective of BIP was to construct a broadcast tree rooted at the source node, and it was built in the way by resembling Prim's algorithm that was used to construct the Minimum Spanning Tree (MST). During each step of BIP, it added one uncovered node with minimum additional cost to the tree. They also suggested a sweep procedure to improve the solution obtained by BIP. The sweep procedure examined each non-leaf node on the tree in ascending order and then reduced the transmission energy if the farthest children can be covered by transmission from the neighbors.

Sausen *et al.* [8] presented an analysis of several solutions for broadcasting. At first, they adapted the dynamic power management with scheduled switching modes (DPM-SSM, which allows a node to switch to a sleep state after a packet transmission) to a blind flooding protocol. The Rakhmatov-Vrudhula battery model was used to capture battery capacity recovery by applying the DMP directly. They also implemented a multi-coverage topology control solution for computing the broadcast backbone. Miller *et al.* [9] developed a distributed broadcast protocol which targeted multi-packet broadcast sessions. They used signal strength measurements to determine link costs, and placed the process during the tree construction on the child nodes.

Papageorgiou *et al.* [10] proposed an optimal and a near-optimal broadcast algorithm, based on the multi-cost approach and selected a set of nodes for broadcasting with the following consideration: (1) the set of node residual energies; (2) the transmission powers used by the nodes; (3) the set of nodes that were covered by a specific schedule.

Several schemes based on distance were proposed for broadcasting. DB (distance based) was one of the schemes used to minimize the effects of the broadcast storm problem when disseminating information in wireless networks [2]. It made use of the distance between the source node and the receiver. The main idea of DB was that a node received a broadcast message for the first time would compute the distance to the source node. If this distance was small, the contribution to the dissemination performing this forwarding was negligible, and the message was not rebroadcast. Ruiz *et al.* [11] enhanced the DB approach by minimizing the transmission power every node used for the broadcasting process in order to save energy and reduce the number of collisions. They added energy efficiency features to the DB approach by reducing the transmission power of the source nodes, and analyzed the influence that reducing the transmission power had over other nodes in terms of the number of collisions or the interference level. In addition, they studied the behavior of the algorithm according to the setting of the delay. Another work [12] considered the optimization of broadcasting algorithm for MANETS: EDB, which was an improved version of distance based protocol. A set of parameters that markedly influenced the behavior of EDB were identified, then a multi-objective algorithm, called CellDE, was used to obtain an optimal performance.

Network coding can be used by the intermediate nodes to combine packets before forwarding. Therefore, it can be used for broadcasting to reduce the total number of transmissions. Li *et al.* [13] applied coding methods to deterministic forwarding node selection approaches to gain a reduction in the number of transmissions, focusing on reducing the number of transmissions each forwarding node performs. They proposed two algorithms that relied on local two-hop topology information and made extensive use of opportunistic listening. One was simple XOR-based coding algorithm, and the other was the Reed-Solomon based coding algorithm. Since XOR-based network coding had been applied for broadcasting and the optimal XOR coding set had been proved to be NP-hard, Fang [14] proposed a vertex coloring based approach to solve the optimal XOR decision problem and applied it into the XOR-based coding broadcast algorithm. In [15] the authors exploited the usage of directional antennas to network coding-based broadcasting to reduce energy consumption. They applied network coding to both dynamic and static forwarding node selection approaches and designed approaches for single source/single message issue in network coding-based broadcast application. Yang *et al.* [16] put forward another network coding-based broadcast algorithm, namely R-Code. By introducing a guardian-ward relationship between neighboring nodes, R-Code distributed the global responsibility of reliable information delivery from the original source to those locally selected guardians. R-Code also used a link quality-based MST as a backbone to guide the selection of guardians adaptively and the transmission of coded packets accordingly. Moreover, R-Code prevented guardians from sending duplicated packets with no extra overhead by adopting network-coding technique.

In order to find better solution, some mathematic heuristic approaches were put forwarded for the MEB problem. Das *et al.* [17] presented three different integer programming models which could be used for an optimal solution of the minimum energy broadcast/multicast problem in wireless networks. All the modes assumed complete knowledge of pair-wise distances between the nodes. They used an LP-based branch and bound method for solving the models. The work of [18] presented some advances in computationally approaching optimal or near-optimal solutions to minimum-energy broadcast problem. The computational machinery consisted of an integer programming model, a bounding algorithm, and a strong heuristic algorithm. In addition, it strengthened the complexity results of some previously proposed algorithm. Montemanni *et al.* [19] proposed the Linear Programming-based Evolutionary Algorithm which used linear programming within the operators of an evolutionary algorithm. The algorithm can be used along or as a refinement tool to improve the results obtained by other algorithms. Wen *et al.* [20] proposed a Lagrangian Relaxation approach which constructed the minimum energy broadcast tree by using a path-based mathematical formulation that differed from the previous link-based and node-based approaches. It could obtain lower and upper bounds for the optimal solution. The narrow gap between the bounds demonstrated in the experimental results indicated that the proposed heuristic algorithm could achieve near optimality.

Considering the minimum-energy broadcast problem can be stated as a combinatorial optimization problem, some scholars used swarm intelligence algorithms to solve the minimum-energy broadcast problem. Wu *et al.* [21] developed a genetic algorithm that used permutation encoding to represent a broadcast scheme. The permutation was transformed into an equivalent broadcast scheme by using a decoder. The sweep procedure was proposed to improve the solution obtained by the genetic algorithm. This genetic algorithm used the rank selection scheme, different crossover operators (partially matched crossover, order crossover and position-based crossover) and different mutation operators (swap mutation, shift mutation and insert mutation). Singh *et al.* [22] proposed a hybrid genetic approach to the minimum-energy broadcast problem. They adopted the different crossover operators and mutation operators with the ones used in [21]. They also used two different variations of *r*-shirnk procedure as local search to improve the solution obtained after the application of genetic operators and decoder. In the work of [23,24] the authors considered the minimum-energy broadcast problem with different scenarios. One scenario was that nodes equipped with omni-directional or directional antenna, the other was the transmission powers of node can be chosen from any real value or just a finite set of possible ones. Then they developed an ant colony optimization algorithm using a sophisticated local search procedure to solve the minimum-energy broadcast problem. Hsiao *et al.* [25] proposed an algorithm based on particle swarm optimization for solving the minimum-energy broadcast problem. During the algorithm, a power degree encoding was used and the search process was guided by the velocities toward personal best solutions and neighborhood best solutions. Besides, they analyzed the local search mechanism: *r*-shrink and developed an improved version.

Lou *et al.* [26] proposed a simple broadcast algorithm, which takes advantage of broadcast redundancy to improve the delivery ratio in an environment that has a rather high transmission error rate. They selected the forwarding nodes to retransmit the broadcast message among the 1-hop neighbors of the sender in a way that the sender's 2-hop neighbors were covered and the sender's 1-hop neighbors were covered by at least two forwarding neighbors, which could improve the delivery ratio and reliability, but also bring too much redundancy to the re-broadcasting. Xu *et al.* [27] developed a mechanism called redundant radius, which involved using two transmission radii, to form a buffer zone that guarantees the availability of logical links in the physical network, one for broadcast tree calculation and the other for actual data transmission. With this mechanism, they extended the centralized broadcast algorithm BIP to make a trade-off between improving network coverage and minimizing energy consumption in broadcasting operations.

The known connected dominating sets (CDS) can be used to solve the minimum-energy broadcast problem in wireless sensor networks. In [28] the authors proposed a connected dominating set election algorithm based on multipoint relays. Without knowing the global network topology, the knowledge assumed for a given node was two-hop neighborhood and the list of neighbors that had selected the node as multipoint relay. In [29] the authors focused on the source-independent Multipoint relays (MPR), which could be used for broadcasting that based on CDS. Then they proposed an extended source-independent MPR by using complete 2-hop information to cover each node's 2-hop neighbor set and by extending the notion of coverage in the original MPR. Han *et al.* [30] proposed a simple and distributed algorithm to construct a CDS in wireless ad hoc networks with time complexity *0*(*n*). In this algorithm, the connected dominating set construction was based on the construction of Maximal Independent Set (MIS). The main idea was to find the dominating set, and then connected any pair of 2-hop-away nodes in the constructed MIS by using one of their common neighbors. Inspired by [30], the work of [31] proposed another heuristic algorithm for constructing a connected dominating set. The main process could be divided into three stages: (1) assign a rank for each node and form an ordered list; (2) construct a maximal independent set; and (3) connect the nodes in the MIS. In [32] an enhanced algorithm to construct a connected dominating set was presented. It used a known local algorithm to determine the primary CDS, and then developed a probabilistic pruning process to implement the secondary CDS. Peng *et al.* [33] presented a broadcast scheme based on the concept of connected dominating set in graph theory. The broadcast message propagated along with the CDS and the nodes in CDS rebroadcast the message. However, there were still redundant nodes in with the above CDS that will weaken system performance of the network. In [34], Guha and Khuller proposed two polynomial time algorithms that achieve approximation factors of *O*(*H*(Δ)) are presented, where Δ is the maximum degree, and *H* is the harmonic function. Ruan *et al.* [35] presented one one-step greedy approximation with performance ratio ln Δ+2, where is the maximum node degree. MCDS in [34,35] is a very good approximation to the optimal solution, and we can adopt it as the basis line for the CDS construction process. Tan *et al.* [36] proposed an efficient placement of proxies for hierarchical reliable multicast which is similar to the broadcasting problem.

## 3. Heuristic Algorithm EMCDS

In this section, we introduce a novel EMCDS algorithm to find the minimum CDS for a given connected graph. The process of the proposed algorithm can be divided into two main steps: (1) Construct an ordered sequence list for all nodes in the network with the help of breadth first algorithm, and build MIS with the ordered sequence list; (2) Connect the above MIS by adding some connected nodes and build CDS, then optimize the connected dominating set and obtain the minimum CDS. The symbols used in the algorithm and details for these steps are described as follows.

### 3.1. Symbols and Definitions

Definition 1. The wireless sensor network can be modeled as an undirected graph *G* = (*V*, *E*), in which *V* denotes the set of sensor nodes, and *E* represents the set of edges among them. Assuming the transmission range *r* is identical to all nodes in the network, there is an edge (*i*, *j*) ∈ *E* if their distance is no more than the transmission range *r*.
Definition 2. Let *U* be a subset of *V* for any given wireless sensor network *G* = (*V*, *E*). *U* is an independent set if (*i*, *j*) 蜓 *E* for any given pairs of nodes *i*, *j* ∈ *U*. And each node in set *U* is called as independent node. *U* is not an independent set if any other node is inserted, then *U* is called as the *Maximum Independent Set* (MIS).
Definition 3. Let *C* be a subset of *V* for any given wireless sensor network *G* = (*V*, *E*). *C* is called as a dominating set if every vertex not in *C* is joined to at least one member of *C* by some edges. Nodes in *C* are called as dominating nodes. *C* is also called as the minimum dominating set if no more nodes can be removed. The dominating set *C* is also called as Connected Dominating Set (CDS) if *C* induces a connected subgraph of *G*. The *Minimum Connected Dominating Set* (MCDS) is the one with minimum number of nodes in the set. For convenience, the notations used in this work are summarized in Table 1.

### 3.2. MIS Construction

MIS is the infrastructure while building MCDS. There are already many methods to build the MIS. In this work, we have introduced a novel notation named *ordered sequence list* that plays an important role in the process of constructing the MIS. The details for the ordered sequence list and MIS construction algorithm are described as follows *via* pseudo code (Algorithm 1):
**Algorithm 1.** MIS Construction Algorithm (MISC).
**MIS Construction Algorithm (MISC)**
Input: the wireless sensor network *G* = (*V*, *E*), the source node *src* ∈ *V*;Output: the ordered sequence list *S*.Run the Breadth First Search (BFS) algorithm on the network *G* with *src* as the root; record the node layer according to their distance to the source, and there is only the source *src* in Layer 0;Sequence the nodes in increasing order of the layer, and build list *L*;Check the nodes from Layer 0 to the end, and order them in decreasing order of the node degree, the result is preserved in list *S*.Set the state of all nodes in *S* as *UNMARKED*;Mark node *src* as *INDEPENDENT*, and add it to list *M*;Mark the state of neighbors of node *src* as *COVERED*, and let *l* = 2;*l* = *l* + 1;For each node *i* in *S* and Layer *l*, check the state; if it is *UNMARKED*, change to *INDEPENDENT* and add *i* into set *M*, and change the neighbors of node *i* to *COVERED* if their initial value is *UNMARKED*, and finally add these neighbors to *C_i_*;If there are still nodes with state as *UNMARKED*, go to step *a7*;End.


As we can see from the pseudo code, Lines *a1*–*a3* concern the construction of the ordered sequence list. In Line *a1*, nodes are ordered with the layer/distance to the source via the breadth first search, and these nodes in the same layer are further ordered with node degree. In this way, nodes in the network are sequenced with *a priori* information that is a combination of the layer and node degree. After the ordered sequence list is obtained, we are to build the maximal independent set via Lines *a4*–*a10*, which is similar to the work done in [31].

In this algorithm, we use different state values to mark the roles of nodes in the network. The nodes are initialized as *UNMARKED*. The *INDEPENDENT* notation is used to denote that a node is included as a member of the independent set, and *COVERED* is used to show that the node is connected to a node in the independent set. After all nodes in the network are marked, the algorithm ends and we can obtain the final MIS as list *M*.

### 3.3. EMCDS Construction

Nodes in the MIS shall be connected to provide a solution for the broadcast problem. Here we introduce an EMCDS algorithm in which we firstly build a CDS and then minimize its size by removing some nodes. The pseudo code of the EMCDS construction is described as follows (Algorithm 2):
**Algorithm 2.** Efficient MCDS Construction Algorithm (EMCDS).
**Efficient MCDS Construction Algorithm (EMCDS)**
Input: the wireless sensor network *G* = (*V*, *E*), ordered sequence list *S*, maximal independent set *M*;Output: the connected dominating set *D*.For each node *i* ∈ *S*, traverse each distinct node *j* ∈ *S* according to the priori, and *j* is added into the list *P_i_* if *j* is adjacent to node *i* and closer / in the same layer to the source;Insert all nodes in list *M* to *D* in the same priori order; set the state of *src* as *CONNECTED*, and all other nodes in *M* as *UNCONNECTED*;For each node *i*∈ *S* excluding the source *src*;If the state of *i* is *UNCONNECTED*, change to *CONNECTED*, and then check *P_i_* in the following way: if there exists a node *j* ∈ *P_i_* ∩ *D*, then *j* is selected as the connected node of *i*, and *i* is inserted into *C_j_*; otherwise, select the first node *k* in *P_i_* as the connected node, and *i* is inserted into *C_k_*;If the state of all nodes in *M* is *CONNECTED*, then go to step *b6*, otherwise go to step *b3*;Remove all the leaf nodes on the spanning tree of set *D*;Set the state of all nodes in *D* as *UNMARKED*;For each node *i* ∈ *D* excluding the source *src*;If the state of *i* is *UNMARKED*, change to *MARKDED*, and then check each *j* ∈ *C_i_*; if there exists some other candidate parent node in the above or same layer for each *j*, remove *i* from the dominating set *D*, and change its children list *C_i_*;End.


**Figure 1.**
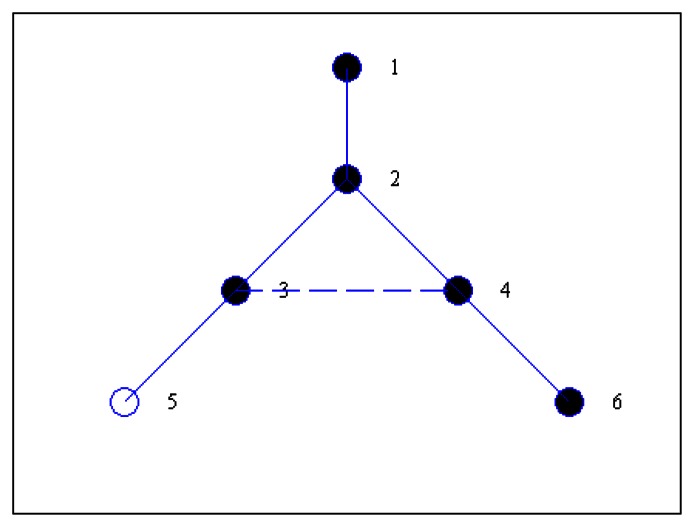
An example network with six nodes.

Lines *b1*–*b5* concern the CDS construction process. Here we use different state values to mark the roles of nodes in the network. The nodes in *M*, except the source node *src*, are initialized as *UNCONNECTED*. The *CONNECTED* identification is used to denoted that a node in *M* has obtained the connected node. Line *b6*–*b10* is the process of optimizing the CDS. Firstly, the state of nodes in *D* is set to *UNMARKED*. After all nodes in the network are *MARKED*, the algorithm ends and we can obtain the final CDS and the MIS as list *M*. Figure 1 shows a simple example to demonstrate result with the above the CDS construction in which the solid circle denotes the selected CDS and lines represents the connection between nodes. Obviously the current *D* is equal to {1, 3, 6, 2, 4}, and the children lists are *C*_1_ = {2}, *C*_2_ = {3}, *C*_3_ = {4, 5}, *C*_4_ = {6} accordingly.

However, with the same example there is still redundancy with the above CDS construction process. For example, node 6 can be removed from the dominating set because it has no children. In the algorithm, we introduce steps to remove such redundant nodes via Lines *b6*–*b10*. The basic idea behind this is that we check whether a node in CDS can be removed by reviewing its children. If all children can find candidate parents in the current CDS, this parent is just removed from CDS. In the following, we also use an example to illustrate the details of the EMCDS algorithm.

### 3.4. An Example Demonstration

**Figure 2.**
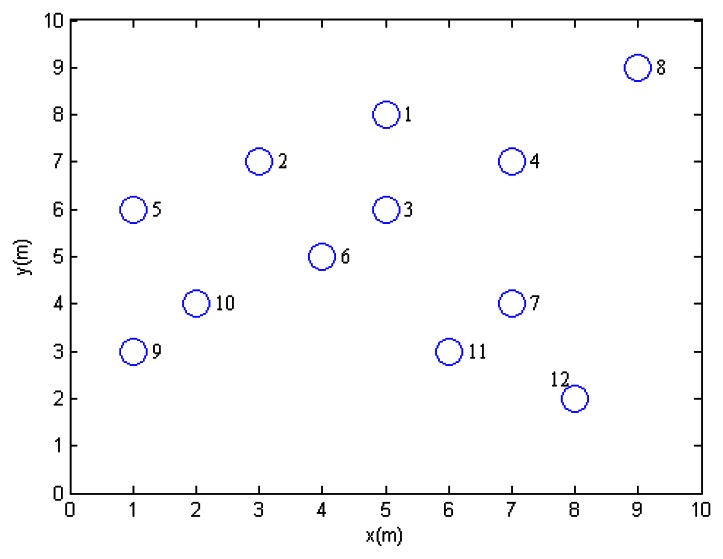
Network topology of the given example.

**Figure 3.**
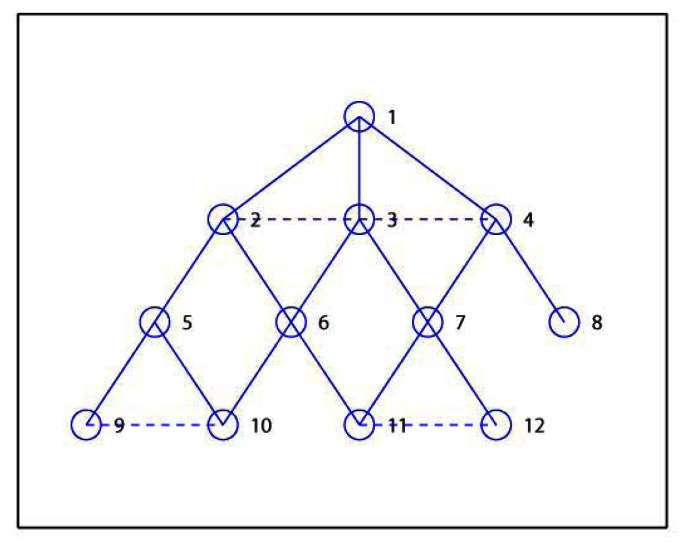
Layers for the given example.

Here we introduce a simple example to demonstrate the process of the proposed EMCDS algorithm. The network is composed with 12 sensor nodes deployed in 10 m × 10 m area. Table 2 provides the coordinates of all nodes in the network and Figure 2 shows the network topology. Assume that the transmission range is 3 and node 1 is the source node. After the broad first search in the MISC algorithm is finished, we can obtain the hierarchical structure for the given network that is illustrated in Figure 3, in which solid lines represent edges between nodes in adjacent layers, and dash lines represents edges between nodes in the same layers. The layer list *L* = {1, 2, 3, 4, 5, 6, 7, 8, 9, 10, 11, 12}, and the ordered sequence list *S* = {1, 3, 2, 4, 6, 7, 5, 8, 10, 11, 9, 12} when ordered sequence is built *via* MISC algorithm.

The next step in MISC algorithm is to build the required MIS. Initially, all nodes are marked as *UNCOVERED* except the source node 1 as *INDEPENDENT*, and thus *M* = {1}. Then the adjacent nodes of node 1, *i.e.*, node 2, 3 and 4, are marked as *COVERED*.

**Figure 4.**
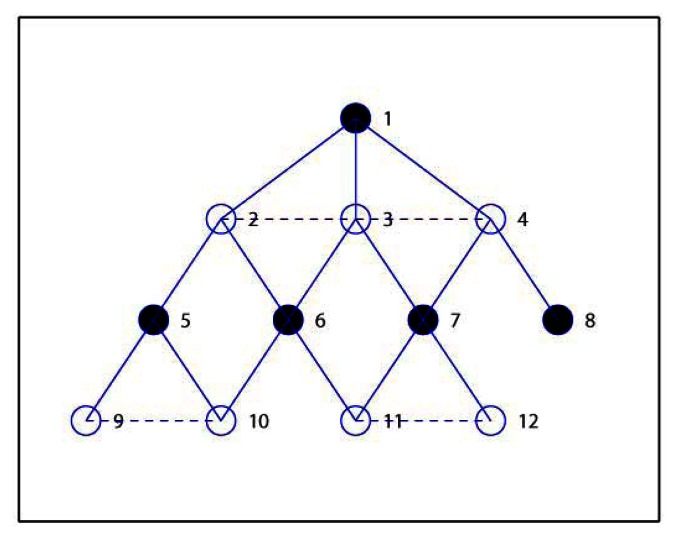
MIS construction for the given example.

Then we check the status of nodes in Layer 2. The node with highest prior in Layer 2 is node 6, which is then marked as *INDEPENDENT* and included in *M* = {1, 6}. The adjacent nodes of node 6, *i.e.*, node 10 and 11 are also marked as *COVERED*, and the children list for node 6 is *C*_6_ = {10, 11}. Then next node 7 is selected, and the similar steps carried out, and finally node 7 is marked as *INDEPENDENT*, *M* = {1, 6, 7}, node 12 is *COVEDED* by 7, and *C*_7_ = {12}. Similarly, in the next step, node 5 and 8 are selected ad marked as *INDEPENDENT*, *M* = {1, 6, 7, 5, 8}, *C*_5_ = {9}, *C*_8_ = Φ. Now we check nodes in Layer 3 and find that they are all covered, and so node 9, 10, 11, 12 are marked as *COVERED*. Finally we have *M* = {1, 6, 7, 5, 8} which is denoted with solid cycles in Figure 4.

**Figure 5.**
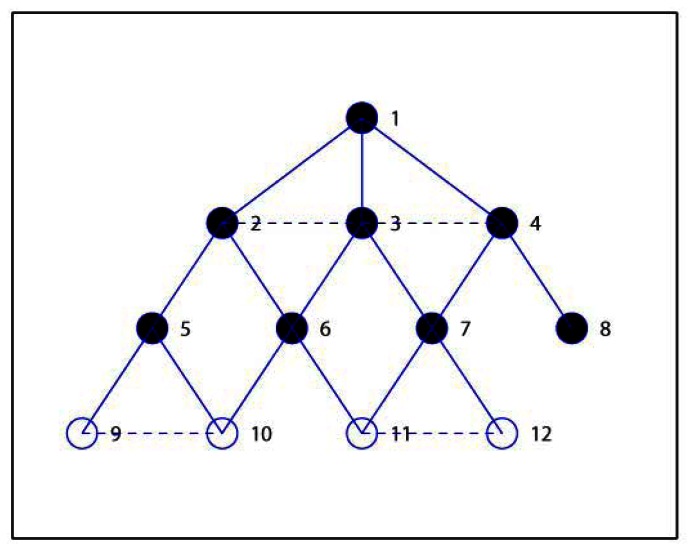
CDS construction for the given example in EMCDS algorithm.

Then we shall add nodes into *M* to construct the required *D* which is initialized equal to *M*. Firstly, we calculate the candidate parent lists for all nodes, and get *P*_2_ = {1, 3}, *P*_3_ = {1, 2, 4}, *P*_4_ = {1, 3}, *P*_5_ = {2}, *P*_6_ = {3, 2}, *P*_7_ = {3, 4}, *P*_8_ = {4}, *P*_9_ = {5, 10}, *P*_10_ = {6, 5, 9}, *P*_11_ = {6, 7, 12}, *P*_12_ = {7, 11}. Secondly, node 1 is marked as *CONNECTED*, and others as *UNCONNECTED*. It can be seen that nodes in *P*_6_ are not included in *D*, and so the first node in *P*_6_, *i.e.*, node 3 is selected as the connected node of 6. Node 6 is also marked as *CONNECTED*, and node 3 is added in the CDS, that is, *D* = {1, 6, 7, 5, 8, 3} and *C*_3_ = {6}. Thirdly, node 3 is the first node in *P*_7_ and is already included in *D*, and so node 7 is marked as *CONNECTED*, *C*_3_ = {6, 7}. Since that node 5 has only one candidate parent as node 2, node 5 is marked as *CONNECTED* with 2 inserted into *D*, *i.e.*, *D* = {1, 6, 7, 5, 8, 3, 2}, *C*_2_ = {5}. In the same way, node 8 is marked as *CONNECTED* with node 4 inserted into *D*, *i.e.*, *D* = {1, 6, 7, 5, 8, 3, 2, 4}, *C*_4_ = {8}. Finally, all nodes are marked *CONNECTED* and the algorithm ends with the final *D* = {1, 6, 7, 5, 8, 3, 2, 4}, as we see from Figure 5.

**Figure 6.**
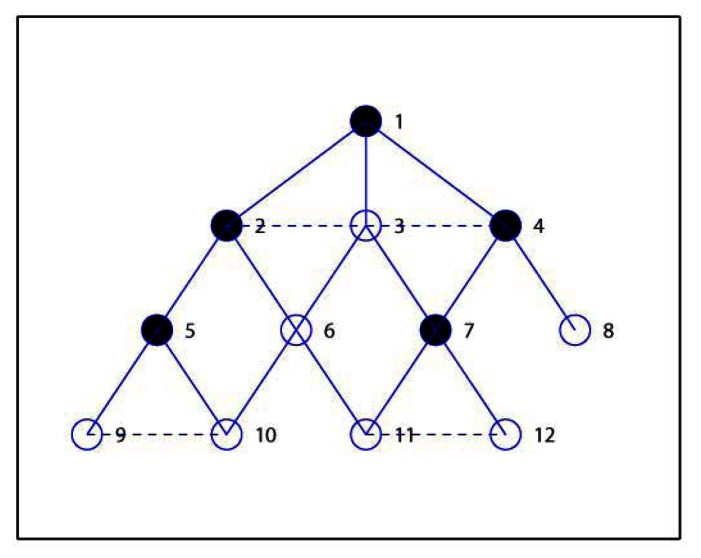
Remove redundant nodes in the EMCDS algorithm.

In the following we illustrate how to use the proposed steps in EMCDS algorithm to remove the redundant nodes from *D*. Firstly, the leaf node, for example, node 8, is removed from the CDS, and thus we have *D* = {1, 6, 7, 5, 3, 2, 4}. In the following we check the existence of nodes whose children are covered by other dominating nodes. It can be seen that node 6 has two children *C*_6_ = {10, 11}, and node 10 can choose node 5, node 11 can choose 7 as their parents. Accordingly, node 6 is removed from *D* and the children lists are refreshed, *i.e.*, *D* = {1, 7, 5, 3, 2, 4}, *C*_5_ = {9, 10}, *C*_7_ = {12, 11}, *C*_6_ = Φ. We check all nodes in *D* in the similar way, and get the final result as *D* = {1, 7, 5, 2, 4}, which is marked as solid cycle in Figure 6.

### 3.5. Complexity Analysis

Theorem 1. The time complexity for the EMCDS algorithm is *O*(*n*^3^).

Proof. We analyze the complexity for each step in the EMCDS algorithm. (1) the time complexity for the breadth-first-search algorithm is *O*(n*e); in the process of building the ordered sequence list, we shall traverse all layers and all nodes in the same layer, it is O(*n*log*n*); (2) We only need to traverse all nodes in the network while construction MIS, and it is *O*(*n*); In the process constructing the connected dominating set, we check each node and their candidate parents, and it is *O*(*n*^2^); Finally we traverse all nodes in CDS and their parents to remove the redundant nodes, and it is *O*(*n*^3^). In this way, the total time complexity of the EMCDS algorithm is *O*(n*e) + O(*n*log*n*) + *O*(*n*) + *O*(*n*^2^) + *O*(*n*^3^) = *O*(*n*^3^).

## 4. MEBS Algorithm

The previous proposed EMCDS algorithm can solve the minimum-energy broadcast problem in wireless sensor networks, in which nodes in MCDS will forward the received messages while nodes excluded in MCDS only need to receive message from their parent nodes. By reducing the number of nodes in MCDS, the algorithm helps to minimize the total energy consumption during the broadcast operation. However, the relay nodes will consume more energy compared with the leaf nodes, and such uneven energy consumption might lead to node failure or network partition. In this section, we first modify the proposed EMCDS algorithm with consideration of the balance of the energy consumption, and then we introduce a new MEBS algorithm to schedule the sensor in sequence for the broadcast operation and aim at providing a scheme with the network lifetime maximized.

The energy efficiency of the broadcast operation concerns severely with the model for energy consumption in the ad hoc sensor networks. Nodes have an initial energy and its value is denoted as *energy*. Let *CostF* and *CostR* be energy consumption for transmission and reception operation and a sensor node, and the previous costs more than the latter [37]. In energy-efficient broadcast problem, nodes in MCDS perform both transmission and reception operations, while other nodes only need to receive the message.

### 4.1. Modified Version of EMCDS Algorithm

Note that in the previous section, the proposed EMCDS algorithm is concerned with finding a CDS with a minimum number of nodes. The balance on the energy consumption shall be further considered to avoid the node failure or network partition problem. We introduce the modification on the previous EMCDS algorithm as follows.

Nodes cannot act as non-leaf nodes if their reserved energy is less than *CostF*. Thus, our previous MISC algorithm shall be modified with the energy considered. Line *a1*, *a3* and *a8* can be improved with the following modification.

*a1*′: Remove the nodes with reserved energy less than *CostF*; Run the Breadth First Search (BFS) algorithm on rest nodes; And finally add each removed node *i* back to the BFS tree by selecting a neighbor as parent which is closest to the root;
*a3*′: If it is the first time to construct the broadcast tree, order all nodes in decreasing order of the node degree; otherwise, order them in random sequence; and the final result is preserved in list *S*;
*a8*′: For each node *i* in *S* and layer *l*, check the state; if it is *UNMARKED*, change to *INDEPENDENT* and add *i* into set *M*, in case that the reserved energy of node *i* is enough to perform the forwarding operation, then change the neighbors of node *i* to *COVERED* if their initial value is *UNMARKED*, and finally add these neighbors to *C_i_*;

Similarly, Nodes cannot act as the connected nodes if their reserved energy is less than *CostF*. Thus our previous EMCDS algorithm shall be modified with energy considered. Line *b4* can be improved with the following modification:

*b4*′: If node *i* is *UNCONNECTED*, change to *CONNECTE*, and then check *P_i_* in the following way: if there exists a node *j* ∈ *P_i_* ∩ *D*, then *j* is selected as the connected node of *i*, and *i* is inserted into *C_j_*; otherwise, select the first node *k* which has enough energy to perform the forwarding operation in *P_i_* as the connected node, and *i* is inserted into *C_k_*.

### 4.2. MEBS Algorithm

The basic idea behind the MEBS algorithm is that we re-construct the broadcast tree if it is necessary. We consider the two special conditions that the broadcast tree is built again as: (1) nodes in the MCDS have spent too much energy on broadcast operation and their total energy runs to a critical point; (2) The reserved energy of the relay node cannot support any forwarding operation. For the first situation we adopt a new parameter *ratio* to denote the ratio between the total current energy and initial reserved energy and for all nodes in the MCDS. In case that *ratio* is less than a given parameter, *i.e.*, *threshold*, it will trigger the MEBS algorithm to re-construct the broadcast tree. The following Algorithm 3 provides details of pseudo code.


**Algorithm 3.** MEBS Algorithm.
**MEBS Algorithm**
Input: Network *G* = (*V*, *E*), *threshold*, *Energy*, *CostF*, *CostR*;Output: Scheduling scheme and the network lifetime.*c1*. Run the modified EMCDS algorithm and get a broadcast tree;*c2*. If the network is connected, go to *c3*, otherwise go to *c6*;*c3*. Use the EMCDS and the broadcast tree for broadcast operation;*c4*. Check the reserved energy of nodes in MCDS, if the energy is less than *CostF*, go to step *c1*;*c5*. Calculate the reserved energy of all nodes in the MCDS as well as *ratio*, if *ratio* is less than *threshold*, go to step *c6*;*c6*. The network ends.


## 5. Simulation Results and Analysis

In this section, we give experimental results for our proposed EMCDS and MEBS algorithms and comparisons with related works. We also analyze the impact of parameters on MEBS. The simulation is done on the Matlab platform.

### 5.1. Simulation Result of EMCDS Algorithm

In this section, we study the performance of our designed EMCDS algorithm compared with other works by experiments. Wireless sensor networks are built with various numbers of nodes and transmission ranges in a 100 m × 100 m square area. When the node position and the transmission range are given, there is an edge between two nodes if their distance is no larger than the transmission range, and then the network *G* is built. Here we use the size of the dominating set to illustrate performance of the algorithm because we aim at minimizing the number of relay nodes in the sensor network in order to reduce the total energy consumption during one single broadcast operation. We consider two scenarios, namely, sparse and dense networks. In each case, the simulation is carried out 100 times and the average performance is analyzed. We compared the EMCDS with the works done by Liao [31] and Ghasemi [32]. It shall be mentioned that to find the optimal solution for MCDS is NP-complete. In this paper, we also adopt the three algorithms in [35,36] which are named as Guha-1, Guha-2 and Roan for easy understanding to describe the basis line of the size of CDS.

**Figure 7.**
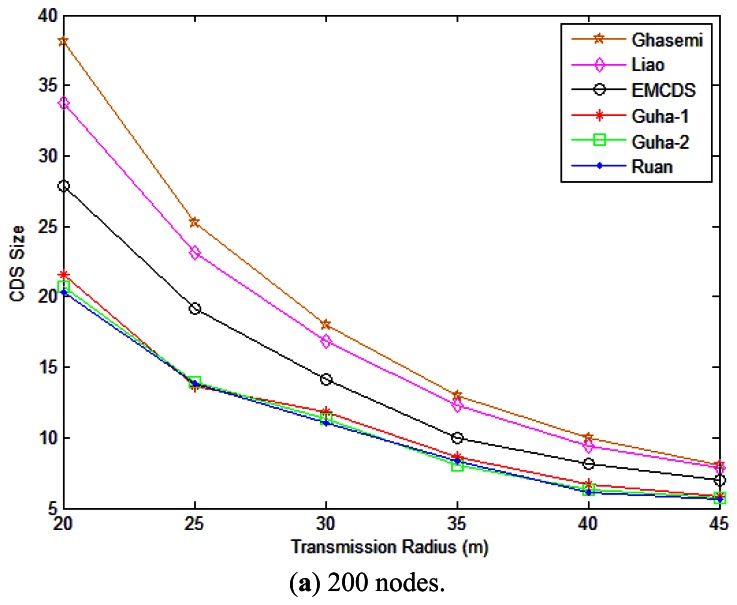

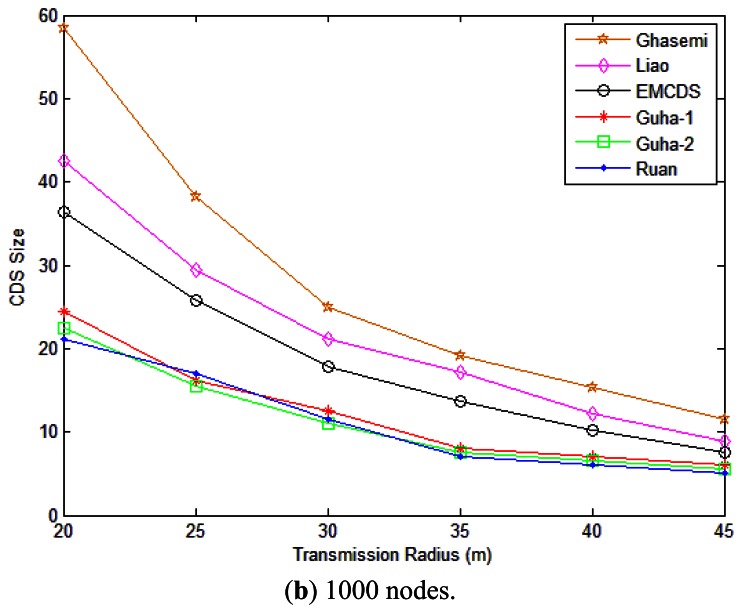
The size of MCDS with different transmission range. (**a**) 200 nodes; (**b**) 1,000 nodes.

For the first scenario, a certain number of nodes (first 200 nodes and then 1,000 nodes) are uniformly distributed in a plane. The transmission range *r* is assumed to vary from 20 to 45 m. As we can see from Figure 7(a and b), the proposed EMCDS has better performance compared with the other two works. For example, in case that node number *n* = 200 and transmission range *r* = 20 m, the average size of CDS set found with the EMCDS is about 26.77% less than that with Ghasemi, and 17.46% with Liao. We also can observe that in case that *n* = 1,000 and *r* = 25 m, the average size of CDS with EMCDS is close to 25.8, while it is 38.2/29.4 with the Ghasemi/Liao algorithms, respectively. An interesting result can also be found with the simulation in Figure 7, namely that the size of CDS decreases with increasing transmission range. This is because more nodes will be covered when the transmission range is increased, and thus the size of CDS is reduced accordingly.

**Figure 8.**
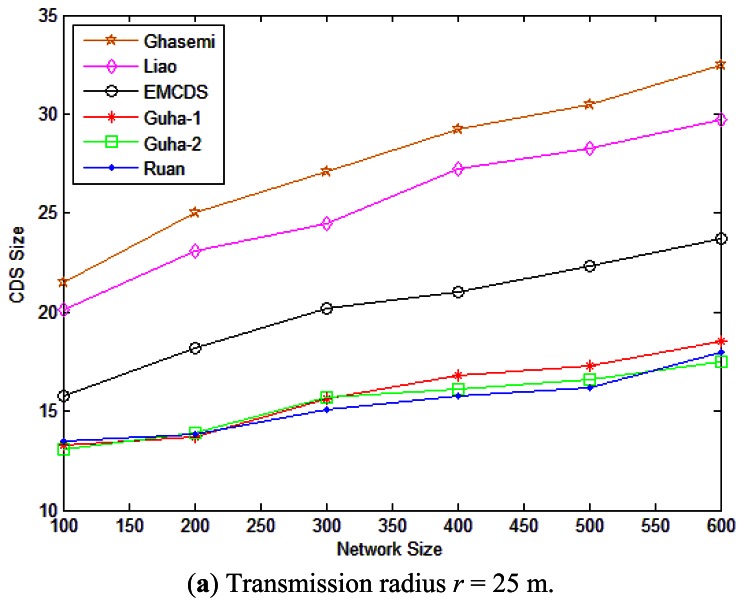

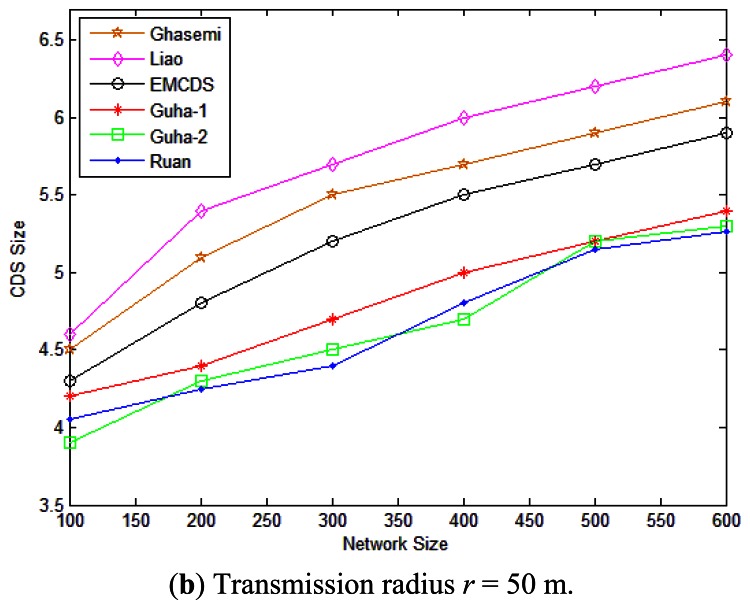
The size of MCDS with different number of nodes. (**a**) Transmission radius *r* = 25 m; (**b**) Transmission radius *r* = 50 m.

For the second scenario, a certain number of nodes ranging from 100 to 600 are uniformly distributed in a plane. The transmission range *r* is set to 25 m and 50 m accordingly. As we can see from Figures 8(a and b), our proposed EMCDS runs better than the other two algorithms. For example, in case that *n* = 500 and *r* = 25 m, the average size of CDS found with EMCDS is about 23.9, while it is 30.5 with Ghasemi, and 28.3 with Liao. In case that *n* = 200 and *r* = 50 m, the average size of CDS with EMCDS is reduced about 5.88% compared with Ghasemi, and 11.11% with Liao.

### 5.2. Simulation Result of MEBS Algorithm

The MEBS algorithm aims at providing a scheduling scheme with the maximized network lifetime. There are a variety of metrics can be used to measure the performance of broadcast protocols, as mentioned in the related works. However, a commonly used metric is the number of message re-transmissions with respect to the number of node in the network. In this work, we use the *saved rebroadcast ratio*, which is initially proposed in [33]. In addition, another important metric is *network lifetime*. The formal definitions of these two metrics are given below:

- *Saved rebroadcast ratio*: Let *n* be the number of nodes that have received the broadcast message and let *tx* be the number of nodes in CDS that actually transmitted the message. The saved rebroadcast ration is defined as (*n*−*tx*)/*n*.
- *Network lifetime*: The network lifetime is defined as the number of broadcast operations carried out before the network cannot be connected.

**Figure 9.**
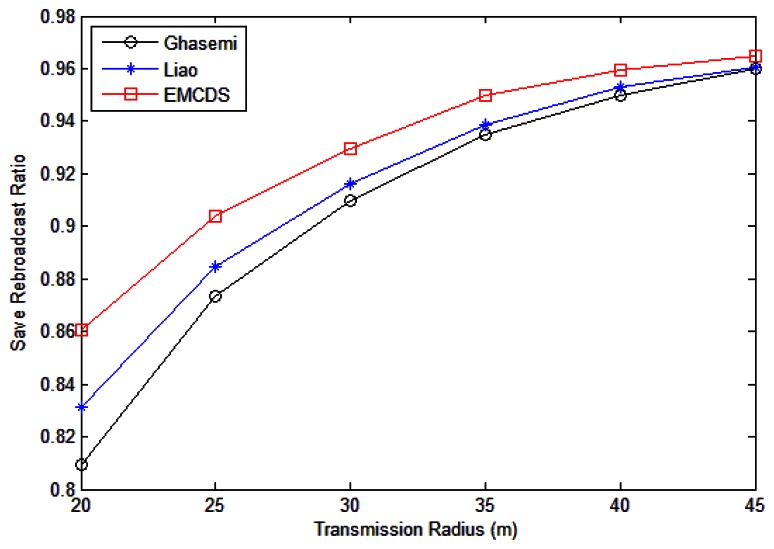
Saved rebroadcast with different transmission range.

**Figure 10.**
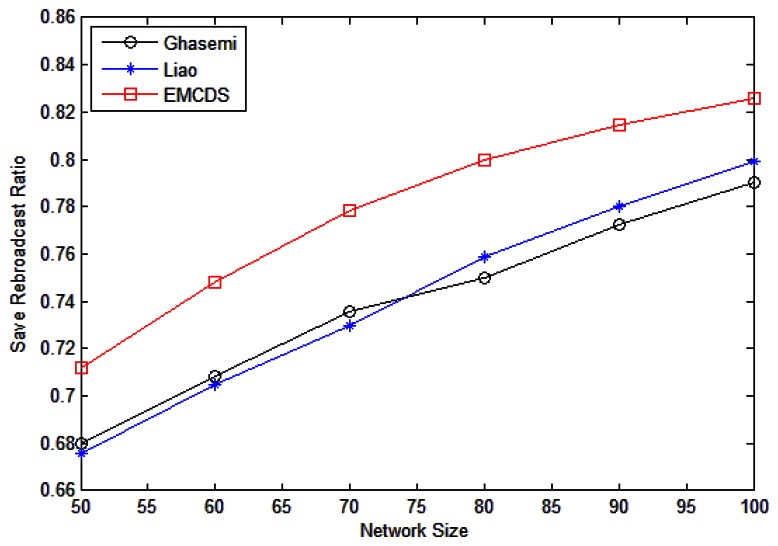
Saved rebroadcast with different number of nodes.

The results for *saved rebroadcast ration* are depicted in Figures 9 and 10. The simulation is done by randomly deploying nodes in an area of 100 m × 100 m. In the following we compare the proposed MEBS algorithm with Liao [31] as well as Ghasemi [32].

Figure 9 has demonstrated the cast the number of nodes is *n* = 200 and the transmission range *r* varies from 20 m to 45 m. As we can see, the saved rebroadcast ratio increases according to the transmission range. It is reasonable because the number of relay nodes reduce while the transmission range is increased. Our MEBS algorithm runs better than the other two algorithms.

Figure 10 illustrates the impact of node density on the save broadcast ratio, in which *r* = 25 m, and the node number *n* varies from 50 to 100. As we can see, the save broadcast ratio with our proposed algorithm is larger than 0.7 in all cases with significant improvement compared with related works. For example, the incremental ratio is about 6.67% compared with Ghasemi, and 5.44% with Liao in case that *n* = 80.

In the following, we will compare the network lifetime via different scheduling algorithms. However, Liao [31] was concerned less with this criterion, and we just modify [31] in the following way to make comparison possible: (1) construct the MCDS with dead nodes ignored; (2) re-construct the broadcast tree with the same conditions as our MEBS algorithm. We also adopt the flooding algorithm as a baseline for the comparison. The simulation is done by randomly deploying nodes in an area of 200 m × 200 m. The original energy for each sensor nodes is assumed to 1,000. The energy cost is 1 for receipt operation, and 4 for transmission, and *threshold* = 0. It means that a node will go to death if the reserved energy is zero, and cannot be relay node if the reserved energy is less than 5.

**Figure 11.**
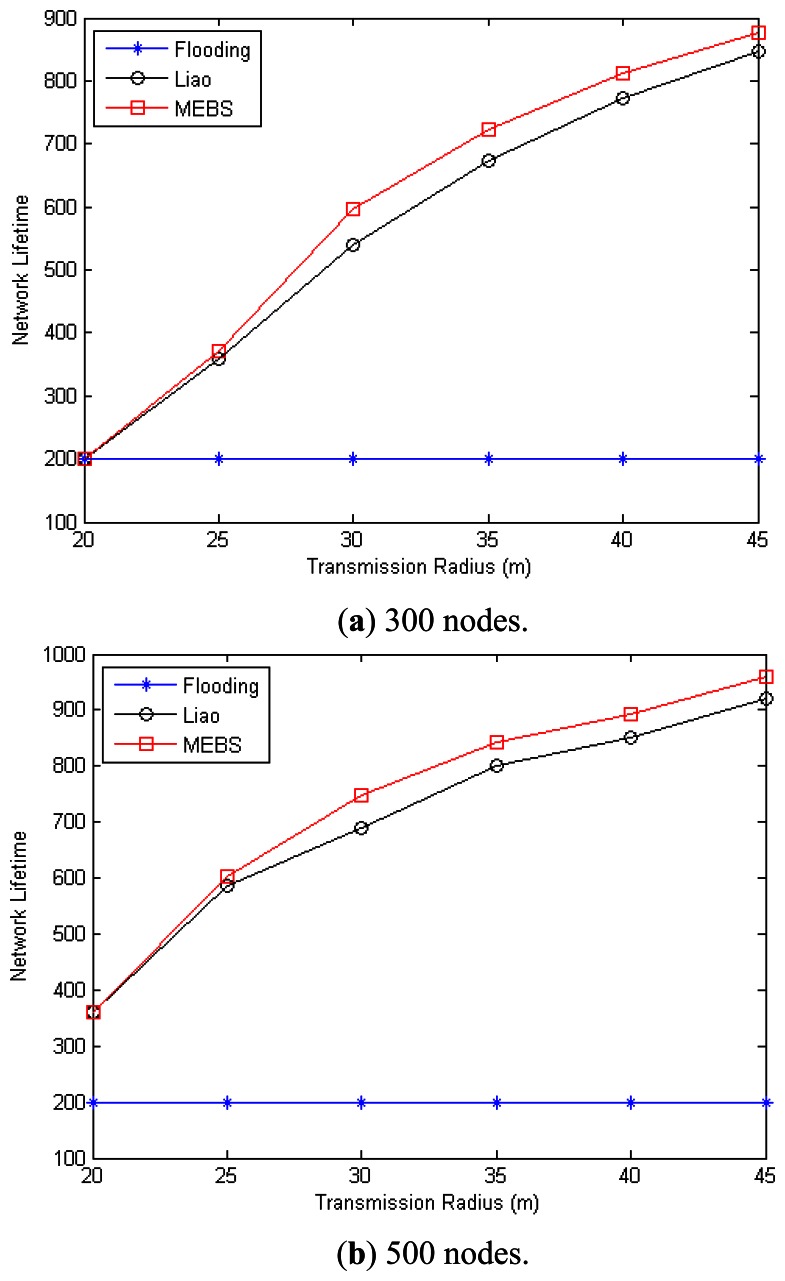
Network lifetime with different transmission range. (**a**) 300 nodes; (**b**) 500 nodes.

Figure 11(a and b) have shown the network lifetime with the transmission range varies from 20 m to 45 m in the case that *n* = 300/500 accordingly. As we can see, the lifetime for the flooding algorithm remains as 200 = 1000/(4 + 1), it is because that all nodes shall forward the received message with the flooding algorithm. In case that *n* = 300 and *r* = 20 m, the network is too sparse and the three algorithms have the same network lifetime. This is due to the fact that some nodes are always selected into CDS in such sparse network, and network is disconnected when these nodes run to death. However, with the increasing of the transmission range, both MEBS and Liao run better than flooding algorithm, and the MEBS is better than Liao. Furthermore, with the nodes increased, the network lifetime increased too. For example, when *r* = 30 m, the network lifetime of MEBS/Liao is 596/538 in case that *n* = 300, and 747/690 in case that *n* = 500.

**Figure 12.**
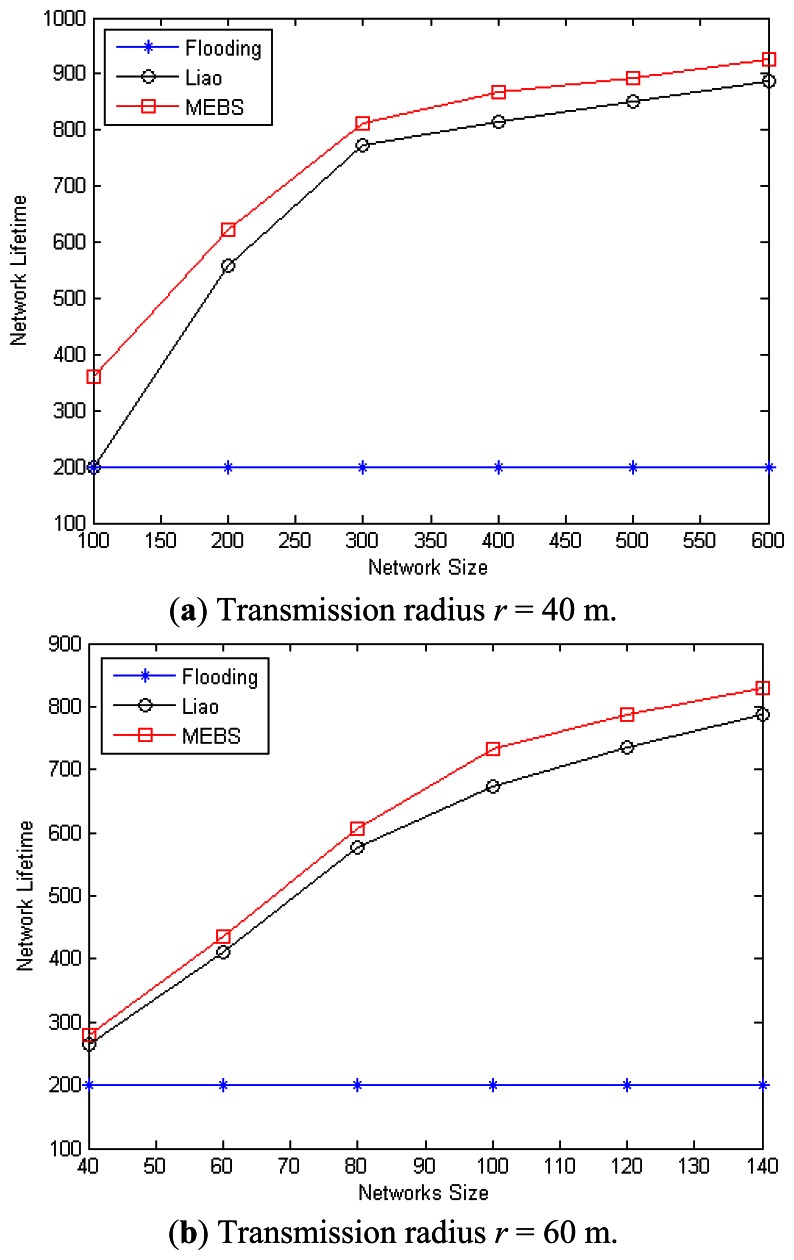
Network lifetime with different number of nodes. (**a**) Transmission radius *r* = 40 m; (**b**) Transmission radius *r* = 60 m.

Figure 12(a and b) have illustrated the impact of node density on the network lifetime, in which the node number varies from 100 to 600 with the transmission range *r* = 40 m and *r* = 60 m, respectively. As we can see, the performance of MEBS is improved a lot compared with the other two algorithms. For example, in the case that *n* = 100 and *r* = 40 m, the lifetime with MEBS is 360, but the others are both 200. In case that *n* = 100 and *r* = 60 m, the network lifetime is increased about 8.9% compared with Liao, and 266.5% with Flooding. It is because that MEBS selects smaller CDS and improves the network lifetime by energy balance. In conclusion, our algorithm MEBS has better performance compared with other works in all case.

### 5.3. Impact of Parameters on MEBS Algorithm

**Figure 13.**
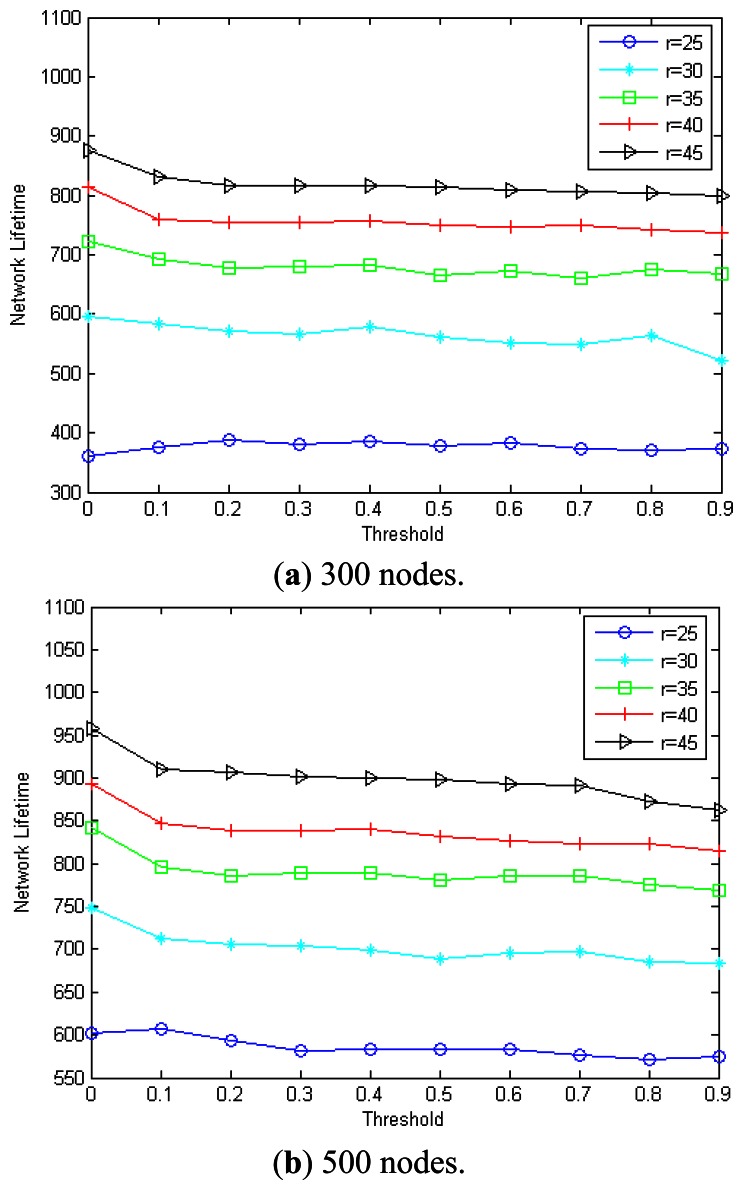
The impact of threshold on the performance of MEBS. (**a**) 300 nodes; (**b**) 500 nodes.

The proposed MEBS will re-construct the broadcast tree according to the parameter *threshold*. In the following we will describe its impact on the network lifetime. The simulation is done by randomly deploying nodes in an area of 200 m × 200 m. Figure 13(a and b) show the impact of *threshold* on the MEBS in different transmission radius with 300 nodes and 500 nodes, respectively. As we can see, the network lifetime increases with the transmission range for a given *threshold* value. For example, the network lifetime is only 379 when *r* = 25 m, and 560/666/750/813 when *r* = 30/35/40/45 m, when *threshold* = 0.5 and *n* = 300. Also, the network lifetime is 583 when *r* = 25 m, and 688/780/831/897 when *r* = 30/35/40/45 m, in case that *threshold* = 0.5 and *n* = 500. The most important fact is that the network lifetime varies with different *threshold* values. For example, when *r* = 35 m and *n* = 300, the network lifetime is 723/692/678/679/682/666/673/659/674/668 when the *threshold* is 0/0.1/0.2/0.3/0.4/ 0.5/0.6//0.7 / 0.8/0.9.

**Figure 14.**
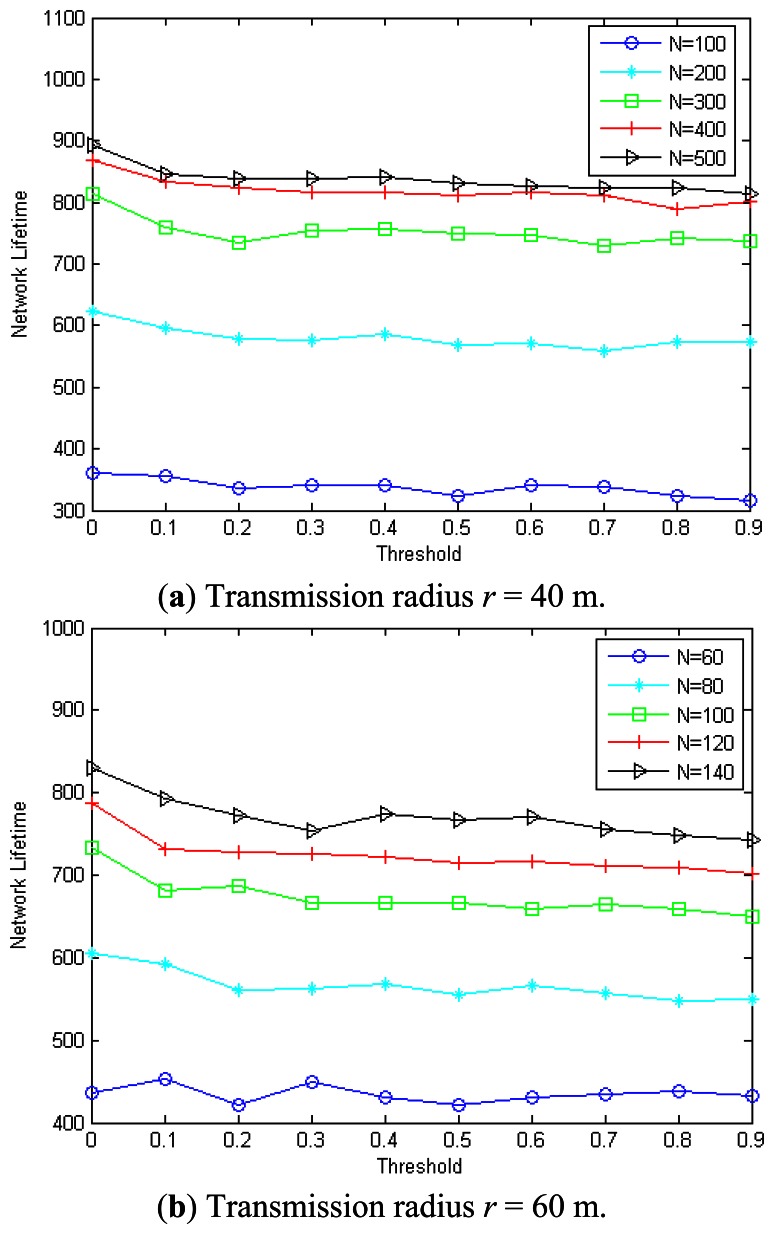
The impact of threshold on the performance of MEBS. (**a**) Transmission radius *r* = 40 m; (**b**) Transmission radius *r* = 60 m.

Figure 14(a and b) show the impact of *threshold* on the network lifetime in different networks with *r* = 40 m and *r* = 60 m, respectively. As we can see, different *threshold* values will lead to different network lifetimes. For example, when *n* = 400 and *r* = 40 m, the maximum network lifetime is 868 with *threshold* = 0, which is about 10.01% better than the minimum network lifetime, that is, 789 when *threshold* = 0.9. We also can observe that the lifetime increases with the node number for a given *threshold*. For example, when *threshold* = 0.1 and *r* = 60 m, the network lifetime is increased about 30.7%/50.7%/61.6%/75.1% when *n* = 80/100/120/140 compared with the case *n* = 60.

Based above observation, we can see that the *threshold* has an important impact on the proposed MEBS algorithm. The network lifetime is always maximized in case that *threshold* = 0 especially in a dense network. For example, when *threshold* = 0, the network lifetime is smaller compared with the other *threshold* in case that *n* = 300 and *r* = 25 m. However, it is not so obvious in sparse networks in which some nodes may always stay in the selected CDS to ensure the network is connected.

## 6. Conclusions

Energy-efficiency is a criterion for sensor nodes and how to perform energy-efficient broadcasting is an important issue in *ad hoc* wireless sensor networks. In this work we are concerned with the minimum-energy broadcast problem and provide a solution from two separate view-points. We have first introduced an efficient heuristic algorithm to build the Minimum CDS (EMCDS) with the help of an ordered sequence list, which aims at building a broadcast tree with minimum-energy cost. To balance the energy consumption in the network, we also have proposed a Minimum Energy-consumption Broadcast Scheme (MEBS) to avoid the node failure problem and aimed at providing an efficient scheduling scheme with maximized network lifetime. The simulation results show that the proposed EMCDS and MEBS algorithms have good performance compared with related works.

In future work, we plan to develop a distributed version of the proposed EMCDS and MEBS algorithms for energy-efficient broadcast problems. Wireless sensor networks are expected to be used in various applications because not only are the sensor nodes rather cheap, but also their robustness and *ad hoc* operation had overwhelmed all other wireless network types. Distributed algorithms are a natural choice for wireless sensor networks. Distributed algorithms should be take advantage of the geometric properties for fast implementation. It is also important to study the impact of limited bandwidth and transceiver resources, as well as to develop mechanisms to cope with mode mobility. Furthermore we will study the energy-efficient broadcast problem in a realistic physical environment in which link failures or delays may occur while one node sends messages to neighbors according to the broadcasting tree, and try to build a test-bed to demonstrate the system performance.

## Figures and Tables

**Table 1.** Notation of the Symbols.

**Symbol**	**Description**
*src*	Sink/source node
*n*	Network size
*L*	Layer list for nodes according to their distance to the source
*l*	Variable used to denote the distance/layer to the source
*r*	Transmission range of the sensor nodes
*S*	Ordered sequence list
*C_i_*	The list of children for node *i*
*P_i_*	The list of candidate Parents for node *i*
*M*	The set for the Maximal independent set
*D*	The set for the connected Dominating set

**Table 2.** Coordinates for nodes in the example.

ID	1	2	3	4	5	6	7	8	9	10	11	12
Axis												
*x*	5	3	5	7	1	4	7	9	1	2	6	8
*y*	8	7	6	7	6	5	4	9	3	4	3	2
